# Editorial: Targeting and monitoring cancer metabolism: novel theranostics for colorectal cancer

**DOI:** 10.3389/fonc.2025.1575563

**Published:** 2025-03-05

**Authors:** Nobu Oshima, Yuki Aisu, Akihiko Masuo, Yoichi Takakusagi, Tatsuya Kawai

**Affiliations:** ^1^ Department of Surgery, Kyoto University Graduate School of Medicine, Kyoto, Japan; ^2^ Department of Surgery, Kobe City Medical Center General Hospital, Kobe, Japan; ^3^ Institute of Quantum Life Science, National Institutes for Quantum and Radiological Science and Technology, Chiba, Japan; ^4^ Department of Radiology, Nagoya City University Hospital, Nagoya, Japan

**Keywords:** cancer metabolism, glycolysis oxidative, phosphorylation, small molecules, imaging technology, 13C-MRI, clinical application

Colorectal cancer (CRC) remains a major global health burden, and there is increasing emphasis on understanding its metabolic landscape and identifying novel theranostic approaches. In recent years, hyperpolarized 13C-MRI, an innovative imaging technique that visualizes the metabolic characteristics of cancer tissue and treatment-induced metabolic changes, has been developed, leading to a surge in research reports on its applications. The advancement of scientific and technological approaches to understanding cancer metabolism has been remarkable, highlighting the rapid progress being made in this field. This Research Topic explores how cancer metabolism can be both targeted for therapy and monitored for disease progression, thereby advancing the field of personalized oncology. The five articles published in this Research Topic collectively make significant contributions to the understanding of metabolic reprogramming in CRC and its potential applications in clinical practice.

The first article, *Multi-omics analyses of glucose metabolic reprogramming in colorectal cancer* (Huang et al.), presents a comprehensive multi-omics approach to dissect the metabolic pathways underlying CRC progression. This study highlights how glucose metabolic reprogramming (GMR) is intricately linked to epithelial-mesenchymal transition (EMT) and metastatic potential. By integrating genomics, proteomics, and metabolomics data, the authors identify novel metabolic markers and propose the CEA/blood glucose ratio as a potential diagnostic tool for CRC liver metastasis.

The second article, *Development and validation of a risk prediction model for sarcopenia in patients with colorectal cancer* (Zhang and Zhu), addresses the often-overlooked issue of sarcopenia in CRC patients. By constructing a predictive nomogram based on clinical and metabolic parameters, the study offers a valuable tool for the early identification of high-risk patients, enabling timely nutritional and therapeutic interventions to improve patient outcomes.

The third article, *Can proline dehydrogenase—a key enzyme involved in proline metabolism—be a novel target for cancer therapy?* (Xu et al.), explores the dual role of proline dehydrogenase (PRODH) in cancer metabolism. While PRODH can induce apoptosis through reactive oxygen species (ROS) signaling, it also promotes tumor survival under hypoxic conditions. This review provides an in-depth discussion of the potential of targeting PRODH as a novel therapeutic strategy for CRC.

The fourth article, *Fer governs mTORC1 regulating pathways and sustains the viability of pancreatic ductal adenocarcinoma cells* (Schrier et al.), investigates the regulatory role of the tyrosine kinase Fer in metabolic signaling pathways. Although the study primarily focuses on pancreatic ductal adenocarcinoma, its findings have broader implications for CRC. The study highlights how Fer-mediated regulation of mTORC1 activity influences cellular metabolism, paving the way for potential cross-cancer therapeutic applications.

Finally *The use of nutrigenomics and nutritional biomarkers with standard care of long-term recurrent metastatic rectal cancer: a case report* (Brinkman et al.), presents a compelling case study demonstrating the role of personalized nutrition in CRC management. By integrating nutrigenomics and monitoring key biomarkers such as folate, vitamin B12, and vitamin D, the study underscores the importance of a multidisciplinary approach to improve long-term patient survival.

Collectively, these articles underscore the evolving landscape of CRC research, emphasizing metabolic targeting and theranostics as essential components of personalized cancer therapy. From fundamental metabolic pathways to clinical applications, this Research Topic provides a foundation for future studies aimed at optimizing therapeutic strategies for CRC.

Regrettably, while this Research Topic provides numerous valuable topics of discussion and insight, it does not include a dedicated discourse on the application of hyperpolarized 13C-MRI. This imaging technique is a crucial element in the development of new cancer therapies, and further research is required to fully realize its potential applications. We look forward to future submissions that introduce novel imaging techniques and fresh perspectives on this technology.

We extend our sincere appreciation to all of the contributing authors, reviewers, and researchers who played a pivotal role in the development of this Research Topic. Their dedication and expertise have made it possible to advance the discourse in oncology, and we look forward to seeing how these findings inspire new research endeavors.

The corresponding author would also like to express gratitude to all contributors who have enriched this theme with their expertise and insights. Finally, thanks also go to co-editors, Professors Yoichi Takakusagi and Tatsuya Kawai, along with Dr. Fuu Oshima, Professors Masayuki Matsuo, Fuminori Hyodo, Neckers Leonard and Murali C. Krishna for the fruitful discussion, Yukako Ito and Sayaka Sugiura for the administrative assistance.

